# Clinical features of multiple myeloma patients with isolated extramedullary relapse

**DOI:** 10.7555/JBR.31.20140090

**Published:** 2015-12-30

**Authors:** Xiaoyan Qu, Lijuan Chen, Tian Tian, Limin Duan, Ruinan Lu, Hua Lu, Hanxin Wu, Jianyong Li

**Affiliations:** Department of Hematology, First Affiliated Hospital of Nanjing Medical University, Jiangsu Province Hospital, Nanjing, Jiangsu 210029, China.; Department of Hematology, First Affiliated Hospital of Nanjing Medical University, Jiangsu Province Hospital, Nanjing, Jiangsu 210029, China.; Department of Hematology, First Affiliated Hospital of Nanjing Medical University, Jiangsu Province Hospital, Nanjing, Jiangsu 210029, China.; Department of Hematology, First Affiliated Hospital of Nanjing Medical University, Jiangsu Province Hospital, Nanjing, Jiangsu 210029, China.; Department of Hematology, First Affiliated Hospital of Nanjing Medical University, Jiangsu Province Hospital, Nanjing, Jiangsu 210029, China.; Department of Hematology, First Affiliated Hospital of Nanjing Medical University, Jiangsu Province Hospital, Nanjing, Jiangsu 210029, China.; Department of Hematology, First Affiliated Hospital of Nanjing Medical University, Jiangsu Province Hospital, Nanjing, Jiangsu 210029, China.; Department of Hematology, First Affiliated Hospital of Nanjing Medical University, Jiangsu Province Hospital, Nanjing, Jiangsu 210029, China.

**Keywords:** multiple myeloma, extramedullary, clinical feature, prognosis

## Abstract

This study sought to analyze the clinical features and prognosis of multiple myeloma with isolated extramedullary relapse and with the absence of systemic progression. The clinical features and outcome were retrospectively analyzed in six multiple myeloma patients. These patients had secretory multiple myeloma at diagnosis. When relapsed, the dissociation between medullary and extramedullary response was detected. The serum or urine monoclonal component was extremely low or absent. The plasma cells in bone marrow were <5%. All patients received new targeted therapies (thalidomide or bortezomib) before extramedullary relapse. It is difficult to achieve second remission for them. Even in those showing response, the duration of response was extremely short. The median of overall survival from diagnosis and from extramedullary relapse was 19 months and 6 months, respectively. The overall survival was significantly shorter compared to the patients without extramedullary involvement (84 months, P= 0.001). These patients exhibited a special and rare relapse pattern. Patients with this relapse pattern were resistant to current therapies, including novel targeted agents and associated with poor prognosis.

## Introduction

Multiple myeloma is a clonal B-cell malignancy characterized by the aberrant proliferation of plasma cells within the bone marrow, as well as at extramedullary sites^[1]^. The neoplastic cells may invade other tissues and organs, such as the liver, lung, spleen, pancreas, kidney and lymph nodes. The digestive tract, thyroid, heart, testis, ovary and skin may also be involved.

Extramedullary disease is a rare primary manifestation of multiple myeloma; however, it appears to increase with repeated relapses^[2]^. Extramedullary neoplasm often occurs with systemic disease at primary diagnosis or the relapse phase^[3]^. Usmani *et al*.^[2]^ reported their experience with extramedullary disease in a large series of 1,965 patients with multiple myeloma. The incidence of extramedullary disease at diagnosis was 3.4% (66 of 1,965). Thirty five patients developed extramedullary disease at the time of relapse or progression.

Extramedullary relapse is one kind of relapse patterns in multiple myeloma^[4]^. It is well-recognized and has been well-documented as early as the 1950's^[5]^. For most patients, extramedullary relapse is accompanied by a wide spectrum of clinical and laboratory abnormalities, such as a marked rise of monoclonal immunoglobulin, free light chain and neoplastic cells within the bone marrow. However, extramedullary expansion of tumor cells can be localized without bone marrow involvement. Therefore, a few patients producing monoclonal immunoglobulins at diagnosis developed extramedullary relapse not accompanied with a parallel increase in immunoglobulins or malignant plasma cells within the bone marrow. In the present study, we identified six Chinese multiple myeloma patients who showed isolated extramedullary relapse with a simultaneous reduction in serum immunoglobulin and aberrant plasma cells within the bone marrow. We studied this distinct relapse pattern from 2007, by thoroughly assessing relevant clinical and laboratory features, prominent similarities, their treatments and response to therapies, mode and speed of progression, and clinical course and prognosis.

## Patients and methods

### Patients

We identified six patients with isolated extramedullary relapse from 213 patients who had been treated at our hospital between December 2007 and November 2013. Initial work-up included bone marrow biopsy and aspiration, skeletal X-ray survey, serum electrophoresis, immunoglobulin quantification, immunoelectrophoresis or immunofixation of serum and urine, complete blood count, measurement of serum creatinine, calcium, lactate dehydrogenase (LDH), β2-microglobulin, C-reactive protein (CRP), and albumin levels, chest X-ray, abdominal ultrasonography, and PET/CT scan when available.

### Assessment of patient response

In this analysis, complete response (CR), very good partial response (VGPR), partial response (PR), stable disease (SD), progressive disease (PD), and relapse were defined according to the International Myeloma Working Group Uniform Response Criteria^[6]^.

### Immunohistochemical staining

Immunohistochemistry was performed on 4 μm formalin-fixed paraffin-embedded sections. Antibodies against the following molecules were used CD38, CD138, CD79, CD20, CD56, and CD3. The percentages of positive cells were scored in 10% increments for each antibody, and the highest percentage was recorded for each case.

### Statistical analysis

Survival analysis was performed using the software packages SPSS17.0 version. The data were expressed as mean±SD, except for data that did not have a normal distribution, which were expressed as median (interquartile range). Overall survial (OS) was calculated as the time from diagnosis to the date of death or last contact. OS analysis was performed by Kaplan-Meier method and compared by log-rank test. All statistical tests were two-sided, and *P *values of less than 0.05 were considered to be significant.

## Results

### Clinical and laboratory features

We calculated the extramedullary relapse-only prevalence among a total of 213 patients with multiple myeloma treated in our hospital between December 2007 and November 2013 as 2.8% (6/213). There were four men and two women with isolated extramedullary relapse. Their median age at diagnosis of multiple myeloma was 57.5 years (range 47-69 years). Two patients had IgG, three had IgA and one had light chain type. Two patients had Durie-Salmon (D-S) stage ⅢB multiple myeloma, and all others had D-S stage ⅢA multiple myeloma. Two had stage Ⅰ, three had stage Ⅱ, and one had stage Ⅲ by the International Staging System (ISS) (***Table 1***).

**Fig.1:**
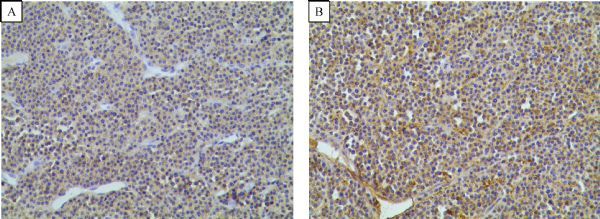
The results of immunohistochemistry for extramedullary involvement were CD38 (+) (A), CD138 (++) (B) (×100), CD79 (+), CD20 (+)/(-), CD56 (-), ki-67 20%-40% (+) and CD3 (-).

The median interval between diagnosis of multiple myeloma and extramedullary relapse was 15 months (range: 10-27 months). Four patients developed extramedullary disease at first relapse and two patients had extramedullary disease after repeated relapses. The extramedullary relapse sites included soft tissue (*n*=3), skin (*n*=1), the liver (*n*=1), soft tissue and central nervous system (CNS) (*n*=1). The number of plasma cells in the bone marrow was less than 5%, while the median number was 33% (range: 12%-61%) at diagnosis. The serum or urine monoclonal component in these patients was extremely low or even absent in one patient. Before extramedullary relapse, one patient achieved CR, three were in VGPR, and two obtained PR. Relapse was diagnosed with imaging techniques including MRI or CT scan in 5/6 (83%). Extramedullary disease was proved by biopsy and the proof of invasion of the CNS was ascertained by positive cerebrospinal fluid cytologic findings. The results of immunohistochemistry for extramedullary involvement were CD38(+) (***Fig. 1A***), CD138(++) (***Fig. 1B***), CD79(+), CD20(+)/(-), CD56(-), ki-67 20%-40%(+) and CD3(-). Other clinical symptoms of extramedullary relapse included deterioration of the patients' general condition in 6/6, renal impairment in 1/6, and recurrence of severe bone pain in 6/6, raised LDH in 6/6, increased β2-microglobulin in 3/6, and elevated CRP in 2/6.

**Tab.1:** The characteristics of patients who developed isolated extramedullary relapse

Pt	Age/Sex	MM type	D-S stage	Time from MM dx to EMR dx (m)	monoclonal Ig at dx (g/L)	PC in BM at dx (%)	monoclonal Ig with EMR (g/L)	PC in BM with EMR (%)	Best response of EMR	alive=1 dead=2	Time of EMR to death (m)
1	47/M	λ	ⅢA	27	3*	20	0.2*	3	PR	2	12
2	59/F	IgG κ	ⅢA	10	42	12	3	3	no therapy	2	3
3	57/M	IgGκ	ⅢA	24	67	35	6	4	SD	1	NA
4	50/M	IgA λ	ⅢB	14	35	61	2.5	2	SD	2	5
5	69/M	IgA λ	ⅢB	16	57	31	8	3	VGPR	2	17
6	58/F	IgA κ	ⅢA	13	50	45	0	0	SD	2	6

EMR: extramedullary relapse; Pt: patient; M: male; F: female; MM: multiple myeloma; EMR: extramedullary relapse; m: month; D-S: Durie‒ Salmon; dx: diagnosis; Ig: immunoglobulin; PC: plasma cell; BM: bone marrow; PR: partial response; SD: stable disease; VGPR: very good partial response; *: urine light chain (g/24 hours); NA: not applicable.

### Clinical course and treatment

All of the 6 patients received novel agents (thalidomide or bortezomib) before extramedullary relapse. Five patients had bortezomib-containing (1.3 mg/m2 day 1, 4, 8, and 11) therapies and five received therapies including thalidomide (100 mg qn). The regimens used before extramedullary relapse were: TAD (thalidomide, adriamycin, and dexamethasone; *n*=1) and VD (bortezomib and dexamethasone; *n*=4), VTD (bortezomib, thalidomide, and dexamethasone; *n*=2); VADT (vincristine, adriamycin, dexamethasone and thalidomide; *n*=2). The median cycles of therapy before extramedullary relapse was eight. After extramedullary relapse, one patient refused further treatment and died three months later. Another patient received therapy including MPR (melphalan, prednisone and lenalidomide) and arsenic trioxide plus vitamin C. He achieved a short response and died of progression 12 months after extramedullary relapse. DCEP (dexamethasone, cyclophosphamide, etoposide, and cisplatin) plus bortezomib and arsenic trioxide plus vitamin C were given to another patient. He did not respond to any anti-myeloma treatment and developed CNS myeloma five months later. Intrathecal chemotherapy with methotrexate, dexamethasone and cytarabine was administered to this patient. He died five months after extramedullary relapse. Another patient, who did not achieve PR after one cycle of DCEP, refused further anti-multiple myeloma therapy. Therapeutic approaches for one patient after extramedullary relapse included VTD (bortezomib, thalidomide and dexamethasone) and DCEP. This patient did not get a second response and survived for six months after extramedullary relapse. One patient achieved VGPR after lenalidomide and dexamethasone therapy. Duration of response and OS from extramedullary relapse of this patient was 14 months and 17 months, respectively.

### Outcome

**Fig.2:**
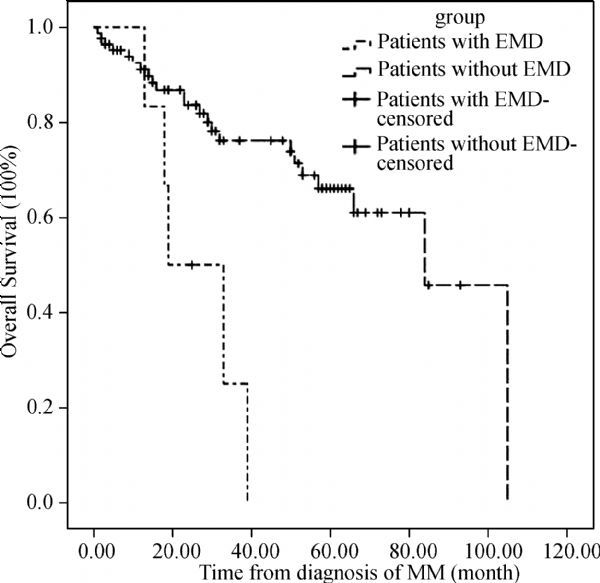
**The overall survival of patients. ** After a median follow-up of 22 months from diagnosis, the median OS from initial diagnosis of patients with EMD and patients without EMD was 19 months and 84 months (P = 0.001). EMD: extramedullary disease; OS: overall survival.

**Fig.3:**
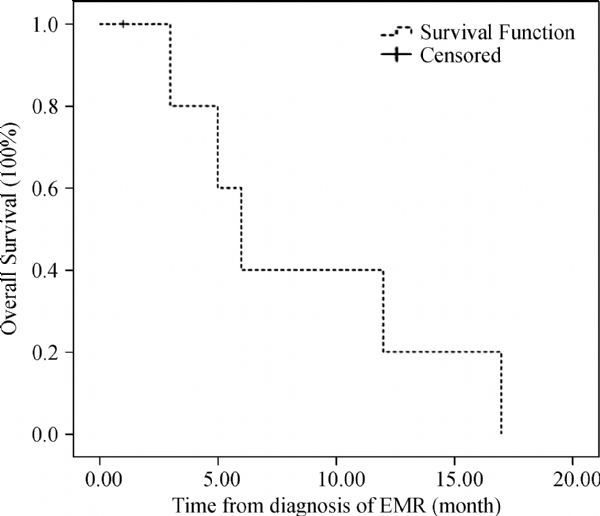
**The overall survival (OS) after isolated extramedullary relapse (EMR).** The median OS after EMR was only six months.

After a median follow-up of 22 months (range: 13-39 months) from diagnosis, the median OS from initial diagnosis and from extramedullary relapse was 19 months (95%CI 4.6-33.4) (***Fig. 2***) and six months (95%CI 3.9-8.1) (***Fig. 3***), respectively. We also analyzed the OS of 85 patients without extramedullary involvement treated in our hospital between December 2007 and November 2013. The median OS was 84 months (95%CI 65.9-102.1) (*P *= 0.001) (***Fig. 2***).

## Discussion

Multiple myeloma is a malignancy characterized by accumulation of terminally differentiated clonal plasma cells mainly in the bone marrow. The extramedullary neoplasm can occur as an extramedullary multiple myeloma with coexisting bone marrow infiltration at primary diagnosis, or at the relapse phase in patients during the course of their disease^[3]^. The mechanisms of extramedullary spread in multiple myeloma are still unclear. Potential mechanisms are decreased expression of adhesion molecules and receptors which are involved in the adherence of myeloma cells to the bone marrow endothelium or the bone marrow homing of myeloma cells^[7^‒^8]^. In this study, we identified six patients who developed unusual relapse just with isolated extramedullary relapse and without systemic progression.

However, these extramedullary relapse-only patients represent an unusual but important phenomenon. We speculated that the myeloma clone of these patients at relapse might be different from the clone at diagnosis. The relapse clone expansion not only became stromal independence but also lost the ability to secrete Ig. Due to the more sensitive imaging technique and prolonged survival, the incidence of extramedullary disease during disease course is rising^[9]^. More attention should be paid toward patients who experienced relapse with dissociation between medullary and extramedullary response.

All of the six patients had received novel targeted treatments before extramedullary relapse. There were several reports discussing the relationship between increased extramedullary relapse and novel targeted therapies. In a study on extramedullary disease in 1,003 consecutive myeloma patients, high-dose therapy or novel agents, such as thalidomide, lenalidomide and bortezomib, did not seem to increase the risk of extramedullary disease^[9]^. However, Short *et al*.^[10]^ studied 174 consecutive patients with relapsed refractory multiple myeloma who were enrolled in a phase II clinical trial of pomalidomide together with low-dose dexamethasone at Mayo Clinic. All 13 patients with treatment-emergent extramedullary disease had previously received novel agents. All of the patients received immunomodulatory agents (thalidomide or lenalidomide); 78% (10 patients) were exposed to bortezomib. Some studies identified not only extramedullary involvement but also biological behavior alterations of myeloma cells in relapsed patients. Dawson *et al*.^[11]^ showed that three patients received novel therapies and showed florid extramedullary relapse with a marked increase in serum free light chain in the absence of a parallel rise in intact immunoglobulin-light chain escape. In addition, it was reported that two multiple myeloma cases developed extramedullary localizations with a concomitant reduction in serum monoclonal protein during bortezomib treatment^[12]^. In accordance with these reports, in this study, all six patients received novel agents before extramedullary disease. Novel biological agents such as thalidomide and lenalinomide are immunomodulators which need the bone marrow microenvironment to exert its anti-myeloma effect. In the context of targeted therapies, de-differentiation of bone marrow plasma cells or alterations in the expression of adhesion molecules on the malignant plasma cell surface may favor the myeloma clone to escape from the bone marrow microenvironment^[13^‒^14]^. Further study is necessary to confirm whether there is a real association between extramedullary disease and novel targeted therapy.

Hitherto, there is no consensus about the best therapeutic choice for extramedullary relapse. A previous study identified that multiple myeloma patients with extramedullary disease at diagnosis received conventional chemotherapy had a significantly shorter survival^[9]^. Several studies showed that thalidomide had lack of efficacy in multiple myeloma patients with extramedullary relapse^[13^,^15^‒^16]^. In contrast to these observations, Biagi *et al*.^[17]^ reported that three patients relapsing with extramedullary disease after hematopoietic autologous stem cell transplant all responded to thalidomide. In contrast to thalidomide, bortezomib produced significant responses in patients with advanced progressive refractory and relapsed multiple myeloma. The efficacy of bortezomib on extramedullary relapse in multiple myeloma has been reported^[18^‒^19]^. However, the number of patients studied at present is small. Case reports have been increasingly showing that lenalidomide was effective on extramedullary relapse^[20^‒^21]^. In our six patients, depending on their initial therapies, we treated two of them with bortezomib and neither showed response. Two patients who received lenalidomide-containing therapy achieved further response. The optimal treatment strategy for isolated extramedullary relapse remains to be defined.

Varettoni *et al*.^[9]^, using a time-dependent analysis, demonstrated that presence of extramedullary involvement at anytime in the course of disease was associated with significantly shorter progression-free survival (PFS) and OS. Even in the era of novel agents, extramedullary disease was associated with a poor prognosis^[2]^. One report studied plasmacytoma relapses in the absence of systemic progression after high dose therapy for multiple myeloma. The natural history of patients with such relapse pattern appears poor. Their median OS was only 12 months. There was an almost invariable progression period to systemic disease with a median of six months^[22]^. A retrospective single-center study of 24 cases demonstrated that the median PFS was two months and the median OS was seven months after diagnosis of extramedullary relapse^[3]^. Fassas *et al*.^[23]^ reported that the median OS from the time of diagnosis of CNS involvement was only 1.5-2 months.

Another study compared the outcome of extramedullary and medullary relapse after autologous or allogeneic hematopoietic stem cell transplant (allo-HSCT) in multiple myeloma. The OS and PFS calculated from the time of relapse or progression after ASCT and allo-HSCT were not significantly different in the bone marrow group as compared to the extramedullary group^[24]^. The median OS of extramedullary relapse alone after autologous stem cell transplant and allo-HSCT was 38 months and 17.3 months, respectively. Our studies showed poor prognosis for isolated extramedullary relapse of multiple myeloma only with 19 months of the median OS and six months of the median OS from extramedullary relapse. The OS was significantly poorer compared with those in patients without extramedullary involvement.

In conclusion, multiple myeloma patients may present dissociation between medullary and extramedullary response, and extramedullary disease may progress despite inconsistent change in serum monoclonal immunoglobulin. Isolated extramedullary relapse of multiple myeloma is extremely rare clinically. However, these patients represent an important pattern of disease relapse. Patients with this relapse pattern were resistant to current therapies including novel targeted agents and associated with a poor prognosis. The number of cases in this study is small, further studies are needed to explore the mechanism of this phenomena and optimal therapeutic strategies to deal with the phenomena.
